# Maternal exposure to antibiotics and risk of atopic dermatitis in childhood: a systematic review and meta-analysis

**DOI:** 10.3389/fped.2023.1142069

**Published:** 2023-05-15

**Authors:** Mengjie Wan, Xiaoyang Yang

**Affiliations:** ^1^ Department of Dermatology, Affiliated Haikou Hospital Xiangya School Central South University and Haikou Municipal Municipal People’s Hospital, Haikou, Hainan, China; ^2^ Department of Hematology, Affiliated Haikou Hospital Xiangya School Central South University and Haikou Municipal Municipal People’s Hospital, Haikou, Hainan, China

**Keywords:** eczema, allergy, antimicrobials, pregnancy, prenatal

## Abstract

**Background:**

Although the association between maternal exposure to antibiotics and the risk of atopic dermatitis (AD) in childhood has been studied extensively, there still is a lack of clarity on the topic. The aim of this study was to summarize the published data and to examine if maternal exposure to antibiotics increases the risk of AD in childhood.

**Methods:**

Systematic search was performed in PubMed, Scopus, Web of Science, and Embase for all types of studies on the review subject independent of any language restrictions and published up to 28th December 2022. Data was analyzed using random-effects model and presented as pooled odds ratio (OR) with 95% confidence intervals (CI).

**Results:**

A total of 18 studies (5,354,282 mother-child pairs) were included. Maternal exposure to antibiotics was associated with an increased risk of AD in childhood (OR: 1.14, 95% CI: 1.06, 1.22, *I*^2 ^= 85%, *p* = 0.0003). The significance of the results was not affected by the location of the study (Asia or Europe). While subgroup analysis based on exposure assessment or diagnosis of AD demonstrated a tendency of increased risk of AD, the association was not statistically significant in multiple subgroups. Segregating data based on the timing of exposure did not affect the significance of the results for studies on all trimesters. However, there was no association between antibiotic exposure in the third trimester or just before delivery and the risk of childhood AD.

**Conclusion:**

The results of this meta-analysis suggest that maternal exposure to antibiotics may lead to a modestly increased risk of AD in offspring. The evidence is limited by high interstudy heterogeneity and bias in exposure and outcome assessment. Future studies are needed to explore if the timing of exposure, the dose, the number of prescriptions, and the type of antibiotic affect this association.

**Systematic Review Registration:**

https://www.crd.york.ac.uk/prospero/, identifier CRD42023387233.

## Introduction

Atopic dermatitis (AD) is a common, chronic, and recurring inflammatory skin disease of childhood. It is characterized by intense itching, dryness, and eczematous lesions (1). Studies show that AD affects around 20% of children, 7%–14% of adult population, and the incidence of the disease has increased dramatically in the last decade due to lifestyle changes (2). AD significantly impacts the quality of life of children and their parents (3). The intense pruritis associated with the disease leads to sleep disturbances, adverse psychosocial effects, and increased financial burden on the patients and on the healthcare system (4).

During the last decade, clinicians focused on identifying risk factors for AD which can be modified to reduce disease prevalence. One such factor is maternal exposure to antibiotics which has been linked with several allergic diseases (5–8). Women's' susceptibility to infections increase during pregnancy and over 40% of women receive antibiotics before the delivery. Recent meta-analyses showed that maternal exposure to antibiotics can lead to an increased risk of asthma and food allergies in childhood (6, 8). Numerous studies suggest that exposure to antibiotics alters maternal microbiota which in turn can influence infant immunity and increase the risk of allergies (9–11).

The relationship between maternal exposure to antibiotics and the risk of AD has been a subject of research in multiple studies (6–8) but with conflicting results. While few studies have reported a positive association (12–14), others have not detected the correlation between maternal exposure to antibiotics and AD (15–17). Several meta-analyses (6–8) have attempted to summarize the evidence. However, all of them had a very small number of included studies, ranging from five to seven. Therefore, the current updated review was designed to examine if maternal exposure to antibiotics is associated with the increased risk of AD in childhood.

## Material and methods

The PROSPERO registration (No. CRD42023387233) of the review was completed before the beginning of the study. The review conformed to the standard instructions of the PRISMA statement (18).

### Literature search

The search strategy was completed independently by two reviewers. Systematic electronic literature search of PubMed, Scopus, Web of Science, and, Embase was done. Since these databases are not comprehensive, a Google Scholar search was also conducted for gray literature. The search was independent of any language restrictions and publication dates, and was completed on 28th December 2022. The terms “eczema”; “atopic dermatitis”; “pregnancy”; “maternal”; “prenatal”; “antimicrobials”; and “antibiotics” formed the various search strings used for the literature search (Supplementary Table S1). The search strings were common to all databases. Two reviewers screened the studies, identified by the systematic search. After removal of duplicates, titles and abstracts of the remaining articles were assessed. Full texts of relevant articles were retrieved and checked for the eligibility by both reviewers. All discrepancies between the reviewers were resolved by discussion.

### Inclusion criteria

The standardized PECOS inclusion criteria were used to select eligible studies. The criteria for each domain were as follows:

*Population*- Mother-child pairs
*Exposure*- Exposure to antibiotics before birth
*Comparison*- No exposure to antibiotics
*Outcome*- AD in childhood (<18 years of age) [Outcome was to be reported as adjusted effect size with 95% confidence intervals (CI)]
*Study type*- All types

Exclusion criteria were: (1) studies not specifically on AD and reporting data on all allergic diseases combined (2) studies not reporting adjusted outcomes (3) studies not reporting exclusively on AD in childhood (4) studies with overlapping or duplicate data. In such cases, the study with the largest sample or the most comprehensive data was selected.

### Data extraction

Two authors extracted the following data in separate forms: author's last name, database of the study, country of origin, study type, sample size, children's age range, method of antibiotic exposure assessment, the timing of exposure, diagnosis of AD, factors adjusted in the data analysis, and effect size. If studies reported multiple ratios of the association for different subgroups, these were combined using the meta-analysis software to generate a single combined ratio. No restrictions were placed on the method of exposure assessment, the timing of exposure, or the diagnosis of AD. All methods reported by the studies were acceptable.

### Quality assessment

Quality assessment was conducted using the Newcastle-Ottawa scale (NOS) (19). Every study was examined for the following: the selection of study population, comparability, and outcomes. These parameters were given a maximum of four, two, and three points respectively. Quality assessment was also carried out separately by the two authors of this review. Any discordant scoring was resolved by consensus.

### Statistical analysis

The meta-analysis was performed using “Review Manager” [RevMan, version 5.3; Nordic Cochrane Centre (Cochrane Collaboration), Copenhagen, Denmark; 2014]. Effect ratios from the studies were combined in a random-effects model to calculate pooled odds ratio (OR) with 95% confidence intervals (CI). The *I*^2^ statistic tool was used for inter-study heterogeneity. Values of >50% indicated high heterogeneity. Funnel plots were used for publication bias. The robustness of the results was checked by excluding one study at a time in a sensitivity analysis. *P*-values <0.05 were considered statistically significant. Subgroup analyses were conducted based on study location, study type, sample size, exposure assessment, diagnosis of AD, and timing of exposure.

## Results

**Figure 1:**
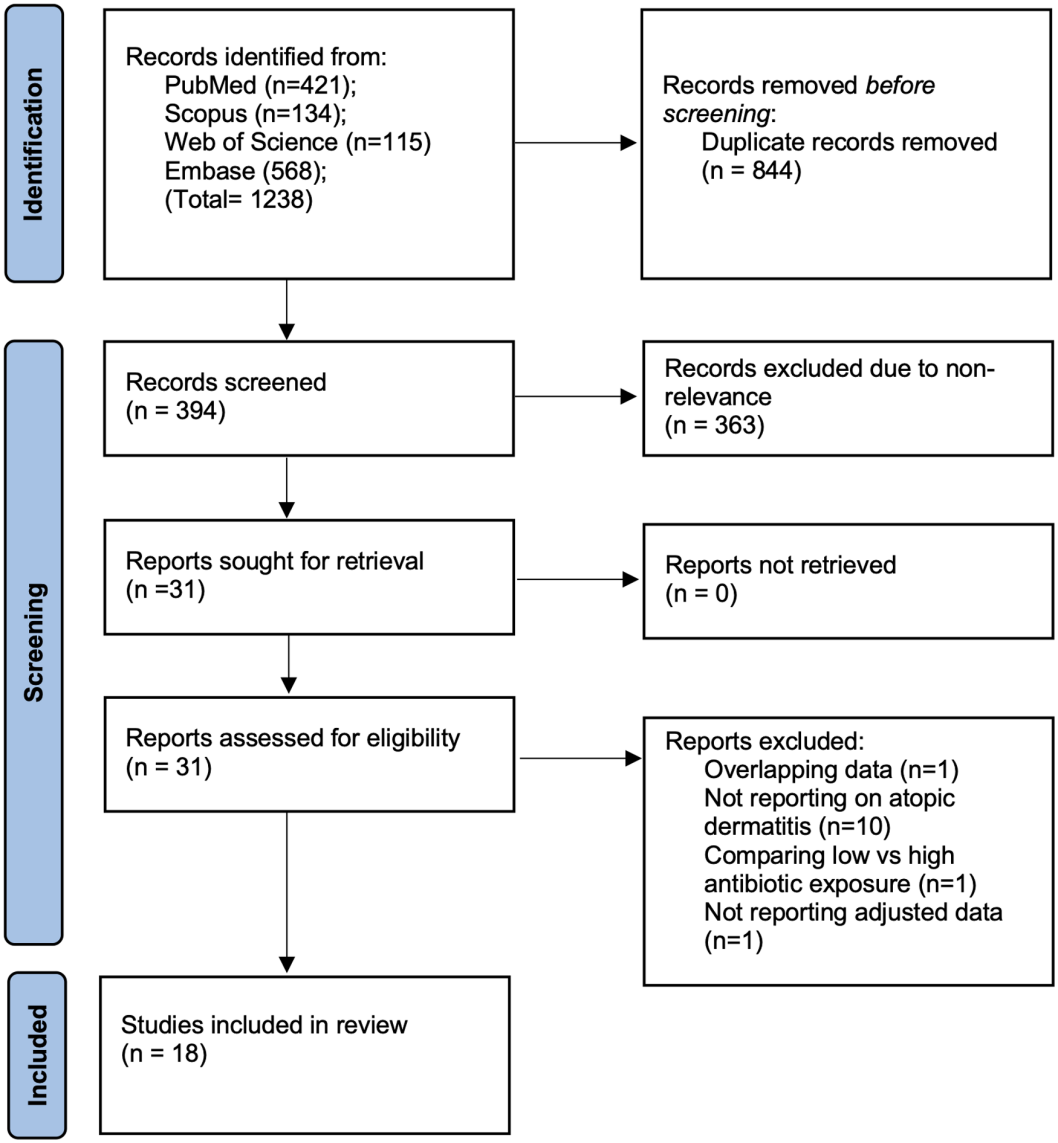
Study flow chart.

Systematic literature search across all databases resulted in 1,238 studies. Of them, 844 were removed as duplicates. Of 394 articles, 31 studies were selected for complete text review. Thirteen studies failed to meet the eligibility criteria. Finally, 18 studies were included in the review (12–17, 20–31) (Figure 1). The inter-reviewer reliability for the selection of studies was high (*κ* = 0.9).

All 18 studies were from Asian or European countries (Table 1). Most of the papers were prospective cohort studies, except for three retrospective cohort studies, two cross-sectional studies, and one case-control study. The studies were published between 2002 and 2022. The total mother-child pair number was 5,354,282. Exposure to antibiotics was determined either by medical records, interviews, and urine samples, or was parent-reported. Most studies did not differentiate the timing of antibiotic exposure. Two studies reported third-trimester exposure, and three studies described pre-delivery exposure. Diagnosis of AD was either clinical or parent-reported or obtained from medical records or interviews. All studies used different confounding variables in the adjusted data analysis. The studies of McKeever et al. (21) and Mubanga et al. (24) used hazard ratios to report the association between antibiotic exposure and risk of AD while all other studies used OR. Due to limitations of data conversion the hazard ratios were pooled in the meta-analysis. The NOS score of the studies was between 6 and 8.

**Table 1:** Details of included studies.

Study	Database	Location	Type	Sample size	Age group	Measurement of antibiotic exposure	Timing of exposure	Diagnosis of atopic dermatitis	Adjusted factors	NOS score
Šumilo et al. (15)	Health Improvement Network and the Clinical Practice Research Datalink primary care databases and the Hospital Episode Statistics database	UK	P	4,461,296	0–5	Medical records	Before delivery	Medical records	Maternal demographic characteristics, behaviour-related factors, pregnancy- and labour-related factors, antibiotic prescribing during pregnancy, maternal long-term allergy- related conditions, deprivation, child demographic and birth characteristics, breastfeeding, child health conditions, and antibiotic prescribing in childhood	8
Stefenaki et al. (25)	Venizeleion General Hospital	Greece	P	385	0–1.5	Parent-reported	All trimesters	Parent-reported	Maternal age, body mass index, smoking, mode of delivery, paracetamol use, parity, type of yogurt	6
Puisto et al. (13)	Ongoing clinical trials NCT00167700	Finland	CC	433	0–2	Medical records	Before delivery	Clinical diagnosis	Original clinical study, child's sex, older siblings, smoking during pregnancy and breastfeeding duration	8
Kelderer et al. (17)	NorthPop birth cohort	Sweden	P	1,219	0–1.5	Parent-reported	All trimesters	Parent-reported	Infant sex, maternal and paternal history of allergic disease, exposure to pets (cats and dogs), and exposure to farm animals	6
Hong et al. (12)	Taixing People's Hospital	China	R	2,909	0–2	Medical records	Before delivery	Assessed by a well-trained investigator	Maternal age, maternal allergy history, parity, gestational age, neonatal birth weight	8
Mubanga et al. (24)	Swedish Medical Birth Register	Sweden	P	722,767	NR (mean age 5.8)	Medical records	All trimesters	Medical records	Sex, birth weight, mother's age, family situation, parity, level of education, area of residence, smoking history, maternal history of asthma, and mode of delivery	8
Geng et al. (23)	Ma’anshan Birth Cohort study	China	P	2,453	0–4	Maternal urine samples	All trimesters	Parent-reported	Delivery mode, parental allergic history, birth weight, mold/dampness in children's sleeping room and current pet-keeping	7
Sasaki et al. (20)	Japan Environment and Children's study	Japan	P	70,408	0–1	Parent-reported	All trimesters	Clinical diagnosis	Maternal education, maternal smoking during pregnancy, maternal history of asthma, atopic dermatitis and allergic rhinitis and child's sex	7
Panduru et al. (22)	Schools and kindergarten	Romania	CS	1,046	0–18	Parent-reported	Third trimester	Parent-reported	Maternal history of atopic dermatitis, parental history of allergy, cesarean section, cat exposure, time of solid food	6
Metzler et al. (16)	European birth cohort study (PASTURE)	Austria, Finland, France, Germany and Switzerland	P	1,080	0–6	Parent-reported	All trimesters	Doctor diagnosis of allergic diseases obtained from questionnaires	Farmer, centre, parental atopic status, gender, smoking during pregnancy, number of siblings, pets during pregnancy, caesarean section, and maternal education.	7
Gao et al. (29)	Streets in Changsha	China	P	903	0–1	Parent-reported	All trimesters	Assessed by a well-trained investigator	Infant's sex, parental history of allergy, maternal age, maternal smoking, maternal education and caesarean section	7
Timm et al. (31)	Danish national birth cohort	Denmark	P	62,560	0–1.5	Interviews	All trimesters	Parent-reported	Prenatal smoking, household socioeconomic, and older siblings	7
Hesla et al. (30)	Assessment of lifestyle and allergic disease during infancy birth cohort	Sweden	R	490	0–2	NR	All trimesters	NR	Lifestyle, living in apartment, living on farm with animals, child room recently painted, pets, time for first body wash	6
Lee et al. (28)	Cohort for childhood of asthma and allergic diseases	Korea	P	412	0–1	Medical records	All trimesters	Clinical diagnosis	Gestational age at birth, sex, pre-pregnancy maternal body-mass index, maternal age at delivery, maternal education level, prenatal exposure to smoke, prenatal exposure to pets, and presence of older sibling(s)	8
Dom et al. (14)	Perinatal factors on the occurrence of asthma and allergies cohort	Belgium	P	773	0–4	Parent-reported	All trimesters	Parent-reported	Gender, maternal age, birth weight, siblings, breastfeeding, pre- and post-natal exposure to cats or dogs, pre- and post-natal exposure to cigarette smoke, day care attendance, parental education and parental history of allergies	6
Bisgaard et al. (26)	Copenhagen prospective study on asthma in childhood	Denmark	P	356	0–3	NR	Third trimester	Clinical diagnosis	Filaggrin gene mutations, mother's eczema, father's asthma and rhinitis, third-trimester alcohol use, longer duration of sole breast-feeding, increases in bedroom temperature, nicotine in hair, presence of dog at birth, and increased baby length	7
Jędrychowski et al. (27)	Kraków birth cohort	Poland	R	102	0–1	Prenatal interview	All trimesters	Standardized interview	Maternal education, child's gender, and maternal allergy	8
McKeever et al. (21)	General Practice Research Database	UK	CS	24,690	0–10	Medical records	All trimesters	Database records	Consulting behavior and maternal allergic disease	8

P, prospective cohort; R, retrospective cohort; CC, case control; CS, cross sectional; NR, not reported; NOS, Newcastle Ottawa scale.

**Figure 2:**
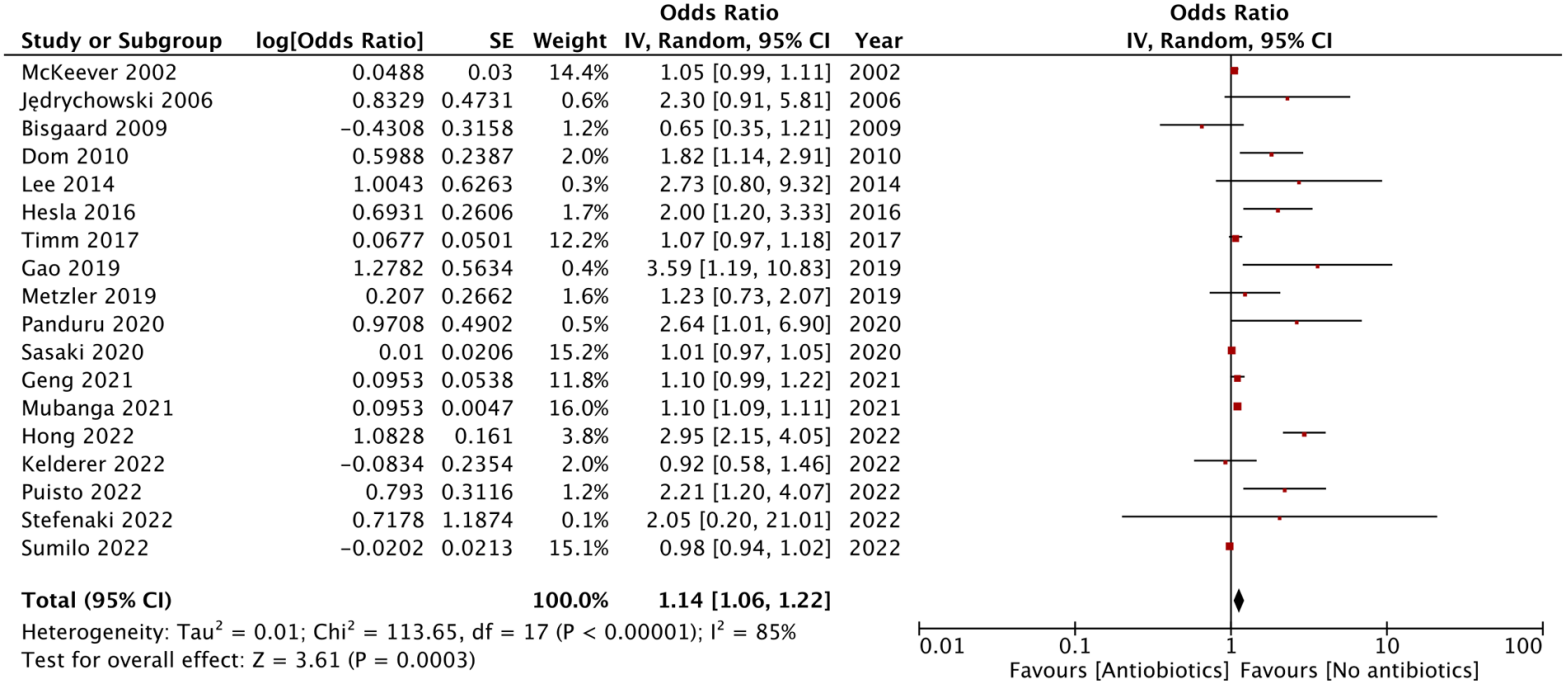
Meta-analysis of association between maternal antibiotic exposure and risk of AD.

**Figure 3:**
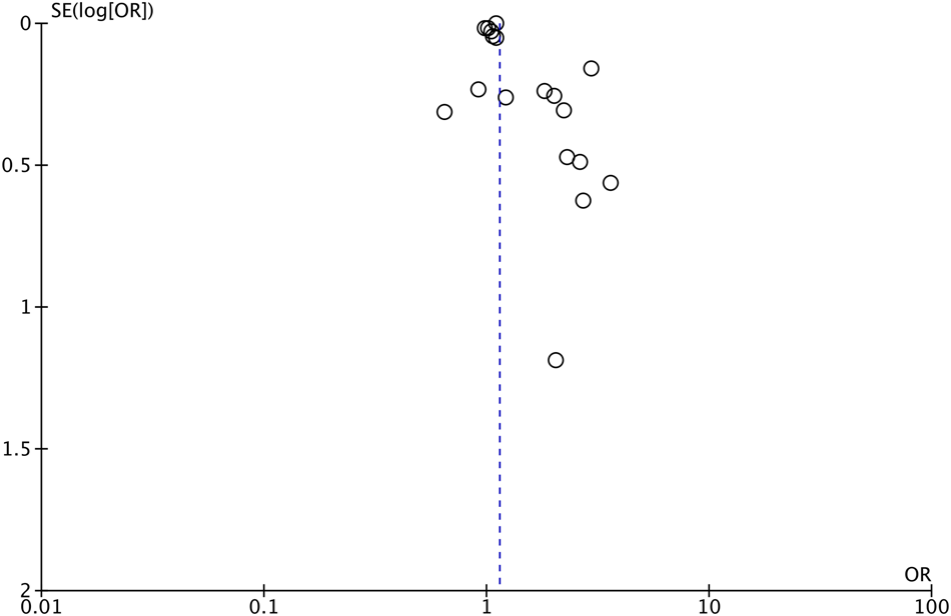
Forrest plot to judge publication bias.

As shown by the results of the meta-analysis of all 18 studies, maternal exposure to antibiotics was associated with increased risk of AD in childhood (OR: 1.14, 95% CI: 1.06, 1.22, *I*^2 ^= 85%, *p* = 0.0003) (Figure 2). The funnel plot did not demonstrate any major asymmetry (Figure 3). There was no change in the significance of results on the exclusion of any study during sensitivity analysis.

The results of subgroup analyses are shown in Table 2. The significance of results did not change based on study location (Asian or European) or sample size of the studies (>1,000 or <1,000). In terms of study type, the results remained significant for retrospective studies but not for prospective or cross-sectional studies. Subgroup analysis based on exposure assessment or diagnosis of AD demonstrated a tendency of increased risk of AD but this increase became statistically insignificant in multiple subgroups. When segregated based on the timing of exposure, the results remained significant for studies on all trimesters. However, there was no association between antibiotic exposure in the third trimester or just before delivery and the risk of childhood AD.

**Table 2:** Subgroup analysis.

Variable	Groups	Studies	Odds ratio
Location	Asian	5	1.53, 95% CI: 1.13, 2.06, *I*^2 ^= 92%
	European	13	1.12, 95% CI: 1.04, 1.22, *I*^2 ^= 62%
Study type	Prospective cohort	12	1.06, 95% CI: 0.99, 1.13, *I*^2 ^= 81%
	Retrospective cohort	3	2.62, 95% CI: 2.03, 3.39, *I*^2 ^= 0%
	Cross sectional	2	1.46, 95% CI: 0.61, 3.48, *I*^2 ^= 72%
	Case-control	1	2.21, 95% CI: 1.20, 4.07
Sample size	<1,000	8	1.81, 95% CI: 1.25, 2.62, *I*^2 ^= 47%
	>1,000	10	1.09, 95% CI: 1.02, 1.17, *I*^2 ^= 90%
Exposure assessment	Interviews	2	1.36, 95% CI: 0.68, 2.72, *I*^2 ^= 61%
	Medical records	6	1.18, 95% CI: 1.06, 1.32, *I*^2 ^= 93%
	Parent-reported	7	1.36, 95% CI: 0.99, 1.87, *I*^2 ^= 62%
Diagnosis of atopic dermatitis	Parent-reported	6	1.13, 95% CI: 0.99, 1.30, *I*^2 ^= 43%
	Medical records	3	1.04, 95% CI: 0.96, 1.13, *I*^2 ^= 93%
	Clinical diagnosis	7	1.64, 95% CI: 0.99, 2.07, *I*^2 ^= 90%
Timing of exposure	Before delivery	3	1.82, 95% CI: 0.78, 4.27, *I*^2 ^= 96%
	All trimesters	13	1.09, 95% CI: 1.03, 1.15, *I*^2 ^= 69%
	Third trimester	2	1.25, 95% CI: 0.32, 4.90, *I*^2 ^= 83%

## Discussion

Over the past two decades, numerous studies have attempted to assess the association between maternal exposure to antibiotics and allergic diseases in children (5–8). Nevertheless, the underlying pathophysiological mechanisms of this association are still unclear. One hypothesis states that the type of microbial exposure received by the infant in-utero is responsible for shaping the intestinal flora and the immune system of the child. Research has shown that microbial colonization in the fetus starts as early as the 11th week of gestation. Therefore, any alteration of microbial flora by antibiotics could increase the risk of chronic allergic diseases (9–11). Using the mouse model, Alhasan et al. (32) showed that exposure to antibiotics during the gestational period correlated with marked dose-dependent changes in maternal and pup microbiota and was associated with a proportionately increasing asthma severity. Another study has reported that maternal exposure to antibiotics led to significant alteration of intestinal microbiota with reduced biodiversity of *Lactobaccillus* and *Bifidobacterium* (33). Both *Lactobaccillus* and *Bifidobacterium* are probiotics. Recent studies showed that early life exposure to these strains can reduce the risk of AD (34). However, the results of the studies in support of this hypothesis are still inconclusive. Corresponding clinical studies have also produced mixed results. While some studies showed that risk of AD increased after maternal exposure to antibiotics (12–14), others found no significant association (15–17).

Considering these lack of consensus, the results of our meta-analysis are of clinical significance, as it was based on a detailed and comprehensive literature search that included higher number of studies compared to previous reviews of Cait et al. (6), Zhong et al. (8), and Huang et al. (7) which included only five, seven, and seven studies, respectively. All three of these previous reviews noted a statistically significant increase in the risk of AD with maternal exposure to antibiotics, with an effect size of 1.28, 1.62, and 1.93, respectively. While our results are in agreement with these studies, we also demonstrate that the risk of AD is very modest at 14% with a narrow CI of 6%–22%. Credibility of our results is further strengthened by their stability, as indicated by the sensitivity analysis.

The primary and most significant limitation of the meta-analysis is its high (85%) heterogeneity. This inter-study heterogeneity may be explained by the varied study populations and methodological differences in the included studies. Multiple subgroup analysis that was performed to assess the source of heterogeneity showed that the results were not affected by the study location and sample size. A slightly increased risk of AD was noted in Asian vs. European studies (53% vs. 12%) but this could be due to a smaller proportion of Asian studies in the review. Also, most of the studies included in the review were predominantly prospective cohort studies. The risk of AD was increased in both study types but just lost statistical significance in the case of only prospective studies.

Assessment of exposure and outcomes can also be a major source of bias. Assessment of antibiotic exposure from medical records and clinical diagnosis of AD is more reliable that self-reported data which can be a subject of recall bias. There is always a possibility of mistaken reporting of drugs used by the women, or reporting other symptoms for AD. However, no marked difference was noted in the subgroup analyses, and all results remained statistically significant or showing a tendency of increased risk of AD.

According to the altered gut microbiota theory, the timing of maternal antibiotic exposure should be an important variable influencing the risk of AD. Theoretically, very early exposure to antibiotics should have minimal impact as there is still enough to restore the maternal microbiota. However, research shows that certain classes of microorganisms, eliminated by antibiotics, are not restored even after nine months (35). Literature is scarce on the association of trimester-wise exposure to antibiotics and the risk of AD. Therefore, our meta-analysis included only few such studies and showed that third-trimester exposure and intrapartum exposure to antibiotics were not associated with an increased risk of AD. These results must be considered in light of changed guidelines that recommend the use of antibiotics before skin incision in caesarean sections (15). However, studies also suggest that the mode of delivery is an important confounder and the correlation between intrapartum antibiotic treatment and AD is increased for vaginally delivered infants but not for infancts, delivered by cesarean sections (12). Similarly, while Mubanga et al. (24) have shown a modest increase in the risk of AD that was associated with maternal antibiotics in all three trimesters, Geng et al. (23) have shown that the timing of antibiotic exposure is confounded by the type of antibiotic. They noted that sulfamethazine exposure in the first trimester and ciprofloxacin exposure in the second trimester increased the risk of AD in offspring. Further research on the timing of maternal antibiotic exposure and the risk of AD is needed.

This review should be interpreted with the following limitations. The pathophysiology of AD is complex with a number of confounding factors such as maternal smoking, socioeconomic status, parental history of allergy, infant gender, birth weight, delivery mode, breastfeeding, pet contact, etc., (7). While our study used adjusted data, there was variability in covariates among studies, and many known and unknown confounders were missed. Secondly, it is possible that the interaction was affected by the multiple variables related to the antibiotic treatment regimen. This review was unable to assess how the timing of exposure, the dose, the number of prescriptions, and the type of antibiotic alter the risk of AD. Thirdly, rigorous recoding of exposure and outcome was not followed in all studies. Medical records could be prone to errors while self-reported data can be erroneous due to recall bias. More objective methods for both exposure and outcome assessment should be utilized by future studies to generate rigorous evidence. Lastly, there was a predominance of European studies in the review and this may affect the general applicability of the results.

In terms of the clinical significance, our review demonstrated that while maternal exposure to antibiotics led to a higher risk of AD in offspring, the increased risk was modest at 14% (6%–22%). Given the high inter-study heterogeneity and bias in the included studies, our results do not recommend avoiding antibiotics in pregnancy. The prescription of antibiotics in pregnancy should be carefully considered after evaluating the risk vs. benefit ratio. Most of the times antibiotics are needed for improving the overall health of the female and prevent any adverse effects of the infectious disease on the fetus. However, mothers should be advised that any intake of antibiotics during pregnancy may lead to a small risk of AD in the child who needs to be closely monitored for early diagnosis and treatment.

## Conclusions

The results of this meta-analysis suggest that maternal exposure to antibiotics may lead to a modestly increased risk of AD in childhood. The evidence is limited by high interstudy heterogeneity and bias in exposure and outcome assessment. Future studies are needed to explore if the timing of exposure, the dose, the number of prescriptions, and the type of antibiotic affect this association.

## Data Availability

Publicly available datasets were analyzed in this study. This data can be found here: The original contributions presented in the study are included in the article/**Supplementary Material**, further inquiries can be directed to the corresponding author.
