# Supplementary material for: Maternal exposure to antibiotics and risk of atopic dermatitis in childhood: a systematic review and meta-analysis
Source: Front Pediatr. 2023 May 15;11:1142069. doi: 10.3389/fped.2023.1142069 (PMC10225666; doi:10.3389/fped.2023.1142069)
Supplement: Supplementary file 1 [file Table1.docx]

Supplementary Table 1: Search strategy

| **Query** | **Search Details** |
| --- | --- |
| (((eczema) OR (atopic dermatitis)) AND (maternal)) AND (antimicrobials) | ("eczema"[MeSH Terms] OR "eczema"[All Fields] OR "eczemas"[All Fields] OR ("dermatitis, atopic"[MeSH Terms] OR ("dermatitis"[All Fields] AND "atopic"[All Fields]) OR "atopic dermatitis"[All Fields] OR ("atopic"[All Fields] AND "dermatitis"[All Fields]))) AND ("maternally"[All Fields] OR "maternities"[All Fields] OR "maternity"[All Fields] OR "mothers"[MeSH Terms] OR "mothers"[All Fields] OR "maternal"[All Fields]) AND ("anti infective agents"[Pharmacological Action] OR "anti infective agents"[MeSH Terms] OR ("anti infective"[All Fields] AND "agents"[All Fields]) OR "anti infective agents"[All Fields] OR "antimicrobial"[All Fields] OR "antimicrobials"[All Fields] OR "antimicrobially"[All Fields]) |
| (((eczema) OR (atopic dermatitis)) AND (pregnancy)) AND (antimicrobials) | ("eczema"[MeSH Terms] OR "eczema"[All Fields] OR "eczemas"[All Fields] OR ("dermatitis, atopic"[MeSH Terms] OR ("dermatitis"[All Fields] AND "atopic"[All Fields]) OR "atopic dermatitis"[All Fields] OR ("atopic"[All Fields] AND "dermatitis"[All Fields]))) AND ("pregnancy"[MeSH Terms] OR "pregnancy"[All Fields] OR "pregnancies"[All Fields] OR "pregnancy s"[All Fields]) AND ("anti infective agents"[Pharmacological Action] OR "anti infective agents"[MeSH Terms] OR ("anti infective"[All Fields] AND "agents"[All Fields]) OR "anti infective agents"[All Fields] OR "antimicrobial"[All Fields] OR "antimicrobials"[All Fields] OR "antimicrobially"[All Fields]) |
| (((eczema) OR (atopic dermatitis)) AND (prenatal)) AND (antimicrobials) | ("eczema"[MeSH Terms] OR "eczema"[All Fields] OR "eczemas"[All Fields] OR ("dermatitis, atopic"[MeSH Terms] OR ("dermatitis"[All Fields] AND "atopic"[All Fields]) OR "atopic dermatitis"[All Fields] OR ("atopic"[All Fields] AND "dermatitis"[All Fields]))) AND ("prenatal"[All Fields] OR "prenatally"[All Fields] OR "prenatals"[All Fields]) AND ("anti infective agents"[Pharmacological Action] OR "anti infective agents"[MeSH Terms] OR ("anti infective"[All Fields] AND "agents"[All Fields]) OR "anti infective agents"[All Fields] OR "antimicrobial"[All Fields] OR "antimicrobials"[All Fields] OR "antimicrobially"[All Fields]) |
| (((eczema) OR (atopic dermatitis)) AND (pregnancy)) AND (antibiotics) | ("eczema"[MeSH Terms] OR "eczema"[All Fields] OR "eczemas"[All Fields] OR ("dermatitis, atopic"[MeSH Terms] OR ("dermatitis"[All Fields] AND "atopic"[All Fields]) OR "atopic dermatitis"[All Fields] OR ("atopic"[All Fields] AND "dermatitis"[All Fields]))) AND ("pregnancy"[MeSH Terms] OR "pregnancy"[All Fields] OR "pregnancies"[All Fields] OR "pregnancy s"[All Fields]) AND ("anti bacterial agents"[Pharmacological Action] OR "anti bacterial agents"[MeSH Terms] OR ("anti bacterial"[All Fields] AND "agents"[All Fields]) OR "anti bacterial agents"[All Fields] OR "antibiotic"[All Fields] OR "antibiotics"[All Fields] OR "antibiotic s"[All Fields] OR "antibiotical"[All Fields]) |
| (((eczema) OR (atopic dermatitis)) AND (prenatal)) AND (antibiotics) | ("eczema"[MeSH Terms] OR "eczema"[All Fields] OR "eczemas"[All Fields] OR ("dermatitis, atopic"[MeSH Terms] OR ("dermatitis"[All Fields] AND "atopic"[All Fields]) OR "atopic dermatitis"[All Fields] OR ("atopic"[All Fields] AND "dermatitis"[All Fields]))) AND ("prenatal"[All Fields] OR "prenatally"[All Fields] OR "prenatals"[All Fields]) AND ("anti bacterial agents"[Pharmacological Action] OR "anti bacterial agents"[MeSH Terms] OR ("anti bacterial"[All Fields] AND "agents"[All Fields]) OR "anti bacterial agents"[All Fields] OR "antibiotic"[All Fields] OR "antibiotics"[All Fields] OR "antibiotic s"[All Fields] OR "antibiotical"[All Fields]) |
| (((eczema) OR (atopic dermatitis)) AND (maternal)) AND (antibiotics) | ("eczema"[MeSH Terms] OR "eczema"[All Fields] OR "eczemas"[All Fields] OR ("dermatitis, atopic"[MeSH Terms] OR ("dermatitis"[All Fields] AND "atopic"[All Fields]) OR "atopic dermatitis"[All Fields] OR ("atopic"[All Fields] AND "dermatitis"[All Fields]))) AND ("maternally"[All Fields] OR "maternities"[All Fields] OR "maternity"[All Fields] OR "mothers"[MeSH Terms] OR "mothers"[All Fields] OR "maternal"[All Fields]) AND ("anti bacterial agents"[Pharmacological Action] OR "anti bacterial agents"[MeSH Terms] OR ("anti bacterial"[All Fields] AND "agents"[All Fields]) OR "anti bacterial agents"[All Fields] OR "antibiotic"[All Fields] OR "antibiotics"[All Fields] OR "antibiotic s"[All Fields] OR "antibiotical"[All Fields]) |
